# Jestings

**Published:** 1866-07

**Authors:** 


### JESTINGS.

Sometimes a narrator of the ludicrous may make a mistake and cause a laugh on the "wrong side of the mouth," and what at one time may be excessively amusing, may at another fail to raise even a smile, for a great deal depends on the state of the mind, but there is scarcely one in a thousand who would not appreciate some one of the following:

"Out of sight, out of mind," but we don't see it; we lost our wallet the other day and it hasn't been out of our mind since.

OLD AND NEW.—"What do Arabs of the desert live on, pa?" asked a roguish girl of her father. "Fudge, Nelly, that's an old conundrum. They live on the *sand which is* (sandwiches) there." "Yes, but, pa, how do they get 'em?" "Well, really, Nelly, you have me there. I give it up." "Why, pa, you know that the sons of *Ham are bred and mustered* in the wilderness?" "Come, come, my daughter, that is too killing; don't say another word." "Oh, yes, do tell me what they eat on their sandwiches." "Eat on 'em; why what do they eat on 'em!" "Butter, to be sure." "Butter? How do they get their butter?" "Why, you know, pa, that when Lot's wife was turned into a pillar of salt, all the family *but her* ran into the wilderness."

—"I will bet you a bottle of wine that you will descend from that chair before I ask you twice." "Done," said the gentleman, who seemed determined not to obey the summons so obediently. "Come down." "I will not." "Then stop till I tell you a second time."

—"What's that ar a picture on?" asked a countryman in a print store the other day of the proprietor, who was turning some engravings. "That, sir, is Joshua commanding the sun to stand still." "Du tell! Which is Josh and which is his son?"

—What is that which is always invisible and never out of sight? The letter I.

—Artemus Ward thus describes his perils at sea: "Deth stared us into the face. But we had rather the advantage of Deth. While Deth stared us into the face, thar was about seventy of us starein Deth into the face. The prospect wasn't pleasin' to us. Not much. I don't know how Deth liked it."

—A lady fixed the following letters in the bottom of a flour barrel, and asked her husband to read them: O-I-C-U-R-M-T.

—Mr. Justice Page was renowned for his ferocity upon the bench. While going the circuit, a facetious lawyer named Crowe was asked if "the Judge was not just behind?" "I don't know," said Crowe, "but if he is, I am sure he was never just before."

—Be temperate in diet. Our first parents ate themselves out of house and home.

—A fashionable but ignorant lady, desirous of purchasing a watch, was shown a very beautiful one, the shop-keeper remarking that it went thirty-six hours: "What, in one day?" she asked.

—"Will you please to help me to some of that butter," said a boarder to another. "It is strong enough to help itself."

—"Ma, is the portrait of father torn?" asked a little cherub of three summers. "No, child, why do you ask?" Why, this morning he said, "Darn my picture!"

—"Many men of many minds," is no longer a truth. A gentleman asked a crowd to imbibe, the other day, with one consent they did.

—A little girl on the cars was asked what motive was taking her to the city. "I believe they call it a locomotive," said the little innocent.

—John Randolph is said, upon one occasion, to have visited a race course near the city of New-York. A flash-looking stranger offered to bet him $500 on the result of the race, and, introducing his companion, said: "Mr. Randolph, my friend here, Squire Tompkins, will hold the stakes." "But, sir, squeaked the orator of Roanoke, "who will hold Squire Tompkins, after I give him my money?"

—A few days since, a gentleman called upon some lady friends, and was shown into the parlor by a servant girl. She asked him what name she should announce, and he, wishing to take them by surprise, replied, "Amicus," (a friend.) The girl seemed at first a little puzzled, but quickly regained her composure, and in the blandest manner possible, observed, "What kind of a cuss did you say, sir?"

—"Can you reach me a potatoe," said a countryman to a gentleman near him at the table, who stretching his fork towards the dish, as if to ascertain the fact, sat down, saying, "Yes, sir," but nary a tater.

—A few weeks after a late marriage, the husband had some peculiar thoughts when putting on his last clean shirt, as he saw no appearance of a "washing." He thereupon rose earlier than usual one morning and kindled a fire. When hanging on the kettle, he made a noise on purpose to arouse his easy wife. She immediately peeped over the blankets, and then exclaimed: "My dear, what are you doing?" He deliberately responded, "I've put on my last clean shirt, and I'm going to wash one now for myself?" "Very well," replied Mrs. Easy, "you had better wash one for me too."

—Mr. Randolph, the celebrated orator and statesman, was lying on a sofa in the parlor of a tavern, waiting for the stage to come to the door. A dandified chap stepping into the room with a whip in his hand, just come from a drive, and standing before the mirror, arranged his hair and collar, quite unconscious of the presence of the gentleman on the sofa. After attitudidizing a while, he turned to go out, when Mr.

Randolph asked him, "Has the stage come?" "Stage, sir! stage" said the fop: "I've nothing to do with it, sir!" "Oh! I beg your pardon," said Randolph, quietly; "I thought you were the driver."

—"I do not give way to puppies," said a man who met Randolph on a narrow foot way; "I do," replied instantly that remarkable man, and, stepping aside, passed on.

—A traveler stopped at an inn to breakfast, and having drank a cup of what was given to him, the servant asked, "What will you take sir, tea or coffee?" That depends upon circumstances," was the reply. "If what you gave me last was tea, I want coffee; if it was coffee, I want tea. I want a change."

—"What do you know of the defendent, Mr. Thompson? Do you consider him a good musician?" "On that point I wish to swear with great care. I do not wish to insinuate that Mr. Vanslopes is not a good musician. Not at all. But I could not help observing (people will observe queer things at times) that after he commenced playing on the clarionet, a saw-filer, who lived next door, left home, and has never yet been heard of."

—A sailor being asked how he liked his bride, replied: "Why, d'ye see I took her for to be only half of me, as the parson says, but dash me if she isn't twice as much as I. I'm only a tar, but she is a Tartar."

—During a recent trial at Auburn, the following occurred to vary the monotony of the proceedings: "Among the witnesses was one, as verdant a specimen of humanity as one would wish to meet with. After a severe cross-examination, the counsel for the Government paused, and then putting on a look of severity, and an ominous shake of the head, exclaimed: "Mr. Witness, has not an effort been made to induce you to tell a different story?" "A different story from what I have told, sir?" "That is what I mean." "Yes, sir; several persons have tried to get me to tell a different story from what I have told, but they couldn't." "Now, sir, upon your oath, I wish to know who those persons are." "Wall, I guess you've tried 'bout as hard as any of them."

—"I've got a new machine," said a Yankee pedler, "for picking bones out of fishes. Now I tell you, its a leetle bit the queerest thing you ever did see. All you have to do is to set it on a table and turn a crank, and the fish flies down your throat, and the bones rite under the grate. Well, there was a country greenhorn got hold of it the other day, and he turned the crank the wrong way; and, I tell you, the way the bones flew down his throat was awful! Why, it stuck the fellow so full of bones that he couldn't get his shirt off for a whole week."

—A young couple had been married by a Quaker, and after the ceremony, he remarked to the husband: "Friend thou art at the end of thy troubles." A few weeks after, the young man came to the good minister boiling over with rage, (his wife was a regular vixen.) "I thought you told me that I was at the end of my troubles?" "So I did, friend, but I did not say which end."

—The report of a court-martial was sent to President Lincoln for his examination, who returned it with this characteristic endorsement: "Whereas, F. W. Smith had transaction with the U. S. Navy Department to a million and a quarter of dollars, and had a chance to steal a quarter of a million; and whereas he was charged with stealing only ten thousand dollars, and from the final revision of the testimony it is only claimed that he stole one hundred dollars, I don't believe that he stole anything at all. Therefore the records of the court-martial, together with the findings and sentence, are disapproved, declared null and void, and the defendant is fully discharged. (Signed) A. LINCOLN."

—An impatient Welshman called to his wife, "Come, come, isn't breakfast ready? I've had nothing since yesterday, and to-morrow will be the third day!" This is equal to the call of the stirring housewife, who aroused her maid at four o'clock with, "Come, Bridget, get up! Here, 'tis Monday morning, to-morrow is Tuesday, the next day's Wednesday—half the week gone, and nothing done yet!"

—A neighbor was telling me of a little boy whose father was a great church-goer, and reverenced the sanctuary. He had been very naughty, and his mother had told him that she was going to tell his father. He quietly retired to the barn, where there was a paint-pot and brush, and painted a church on the seat of his pants, and running to his mother, he says: "O mother! I guess father will not whip me, for I have painted a church on my pantaloons, and he will not whip the church."

—The alderman who was lately injured by the accidental discharge of his duty, is reported to be in a fair way of recovery. He says he'll never be caught that way again while in the full possession of his senses.

—A fashionable party is now called a "daughtercultural show."

—"They say 'cotton is declining,'" exclaimed an old lady, as she removed her spectacles and laid down her paper. "I thought so," she continued, "for the last thread I used was very feeble."

—"I am glad this coffee don't owe me anything," said a bookkeeper to his wife the other morning at breakfast. "Why so?" was the question. "Because I don't believe it would ever settle."

—HIBERNIAN TOASTS.—Two gallant "sons of Erin," being just discharged from service, were rejoicing over the event, when one, who felt all the glory of his own noble race, suddenly raised his pot above his head, and said, "Arrah, Mike, here's to the gallant old 69th—the last in the field, and the first to leave it." "Tut, tut, man," said Mike; "ye don't mane that." "Don't mane it, is it?" Then what do I mane?" "You mane," said Mike, and he raised his glass high, and looked lovingly at it, "Here's to the gallant 69th, equal to none!" And so they drank.

—A learned and enthusiastic orator recetly startled his audience by the following sentence: "Sir, let those beware who would trifle with the popular will. In the language of the poet, '*Facilis descensus averni*'—The voice of the people is the voice of God." This is nearly as good as the illustration once used by a member of a certain Down East Legislature: "Mr. Speaker," said the member, referring to the question under debate, "this matter is like Pandora's box—the more you stir it, the worse it smells!"

—A youth, who much desired to wear the matrimonial yoke, had not sufficient courage to pop the question. On informing his father of the difficulty he labored under, the old gentleman replied, passionately, "Why, you great booby, how do you suppose I managed when I got married?" "Oh, yes, you married Mother, but I've got to marry a strange girl!" said the bashful lover.

—How many rods make an acre?" a father asked of his son, a fast urchin, as he came home one night from a town school. "Well, I don't know, governor," was the reply of the young hopeful, "but I guess you'd think one rod made an acre, if you'd got such a tanning as I did from old vinegar face this afternoon."

—The owner of a large dog at Grand Rapids, Mich., a few days ago, placed a hundred dollar looking-glass before his canine to worry him. The dog flew round barking and growling. The owner was delighted and cried "seek 'em!" The dog "seeked," and the mirror and the "other dog" disappeared at at the same time.

—The editor of *The Woonsocket Patriot* makes merry over the mistake of an old Shanghai hen of his that has been "setting" for five weeks upon two round stones and a brick! "Her anxiety," quote he, "is no greater than ours to know what she will hatch. If it proves a brickyard that hen is not for sale.

—A young lady went into a store to buy a piece of music, entitled, "When I sleep I dream of thee; but by a ludicrous mistake, she astonished the young man by inquiring if he had the music entitled, "When I dream I sleep with thee."

—A down-east Yankee, seeing an alligator for the first time on the Mississippi, with his mouth open, he exclaimed: "Well, he ain't what you may call a hansum critter. But he's got a good deal of openness when he smiles.

—A man out West says he moved so many times during one year that whenever a covered wagon stopped at the gate his chickens would fall on their backs and hold up their feet, in order to be tied and thrown in.

—A Shilling Temptation.—A young man in England, having entertained a tender passion for a young woman, felt such insurmountable diffidence as to prevent his ever disclosing the same to the fair empress of his heart, and resolved on an expedient which would bring the business to an issue. He went to the clergyman, and requested the bans of marriage might be published according to law. When the publication was brought to her ears, she was filled with astonishment, and went to him to vent her resentment; he bore the sally with fortitude, observing that if she did not think proper to have him, she could go to the clergyman and forbid the bans. After a moment's pause, she took counsel with her anger, and said: "Well, as it has been done, it is a pity that the shilling should be thrown away!

—A little ragged urchin, begging in the city the other day was asked by a lady who filled his basket, if his parents were living? "Only dad, marm," said the boy: "Then you have enough in your basket now to feed the family for some time," said the lady. "Oh, no, I haven't neither," said the lad, "for dad and me keeps five boarders; he does the house work, and I do the market'n."

—NEVER MIND THE WOOD SHED.—"My dear Amelia," said a dandy, "I have long wished for this opportunity, but hardly dare speak now for fear you will reject me. But I love you; say you will be mine! Your smiles would shed—" and then he came to a pause; "your smiles would shed—" and then he paused again. "Never mind the wood shed," said Amelia, "go on with the pretty talk."

—A young Miss who was visiting in a neighboring town recently received a letter from a friend at home, in reply to one which she had made a request to be told "all the news." Her friend wrote: "I have not much to write to you, but the ladies' aid society meets at our house next week, when I shall hear the news about everbody in town."

—Which has most legs, a horse or no horse? No horse has five.

—If a toper and a gallon of whisky were left together, which would be drunk first?

—When do you indulgein your most extravagant repast? When you have a piano for tea (forte).

—Why is a heartless kiss like a stage on a cold day? Because it's a 'bus with no warmth in it.

—A gentleman, one evening, was seated near a lovely woman, when the company around were proposing conundrums to each other. Turning to his companion, he said, " Why is a lady unlike a mirror? She " gave up." "Because," said the rude fellow, " a mirror reflects without speaking; a lady speaks without reflecting." " Very good," said she. " Now answer me. Why is a man unlike a mirror?" " I cannot tell you." " Because the mirror is polished, and the man is not."

—The man who takes things easy---the pickpocket.

---A soldier with a huge pair of understandings called upon a boot black the other day to shine up his boots. The urchin contemplated the size of the job for a moment, and then called out to a comrade, " I say, Bill, lend us a spit, won't yer? I've got an army contract."

---" I'll bet a sheep," said old Meredith to his better half, " that our boy Otho is goin' crazy: fur he's grinnin' at the plow, he's grinnin' at the barn and he's grinnin' to himself wherever he goes." "Sho! old man," said his wife, "you don't know nothin." The critter's got a love letter."

---A little shaver was sent to an undertaker, to order a coffin for an infant. " How old was the child," asked the undertaker. " It was not old at all," was the answer, " it died a bornin." This " plan" of the reconstruction committee has met the same untimely fate.

---Sensible Advice.---An exchange paper among other suggestions which will enable a person to avoid the cholera, says: " Endeavor, if possible, to keep a clean conscience, and two or three clean shirts. Rise with the lark, but avoid larks in the evening. Be above ground in all your dwellings, and above board in all your dealings. Love your neighbor as yourself, but don't have too many in the same house with you.

-----We trust the Lord is on our side, Mr. Lincoln," said the speaker of a delegation of Christian men to that good man, during one of the darkest days of the rebellion. " I do not regard that so essential as something else," replied Mr. Lincoln. The pious visitor looked horror struck until the President added: " I am most concerned to know that we are on the Lord's side."

—Some papers having made the statement that butter should not be kept in the same room with kerosene oil, as the kerosene would spoil the butter—giving it a peculiar flavor—the Elmira *Advertiser* remarkes that some of the butter now-a-days is enough to spoil kerosene.

—A Kentuckian becoming incensed at the boastfulness of an Englishman in the train of Sir Morton Peto, as to the superiority of British inventions, exclaimed, "Pshaw! *They* are of no account. Why a house painter in my neighborhood grained a door so exactly in imitation of oak, that last year it put forth leaves, and grew an excellent crop of acorns; and another fellow up in Iowa has just taught ducks to swim in hot water, and with such success that they *lay boiled eggs!*" The Englishman from that time forth exhibited a modest and subdued air.

—A flippant young man observed, in the presence of Dr. Parr, that he never believed any thing he could not understand. "Then yours must be the very shortest creed of any man's I know," remarked the doctor.

—A little girl—an only child—one day looked up in her mother's face with "Mother what do you make me wear this old frock for? I should think you might afford to dress me better seeing as how there's *only one of us.*"

—Brigham Young is indeed a pillar of Salt Lake. His idea of a wife is—*Lots.*

—A woman is like ivy—the more you are ruined the closer she clings to you. A vile old bachelor adds: "Ivy is like a a woman—the more it clings to you, the more you are ruined." Poor rule that won't work both ways.

—"Frank," said an affectionate mother the other day to a promising boy, "if you don't stop smoking and reading so much you will get so after a while that you won't care anything about work." "Mother," replied the hopeful leisurely moving a very long cigar, "I have got so now."

—Said a gentleman to another in a bar-room, "Who is that ugly woman with whom you were just walking?" "That is my sister." "No, no, I don't mean her," explained his unfortunate interrogator, "I mean that horrid, red-haired creature by her side." "That, sir, is my wife."

—"I tell you," said a warm friend of a newly-elected Senator, to an old sober politician, "your party may say what they please, but cannot deny that Mr. C—— is a sound man." "That's just what we are afeared on," replied old Beeswax: "it's our opinion that he's all sound!"

—Said a little four year old, "Mother! Father won't be in heaven mith us, will he?" Why, my child? "Because he can't leave the store."

—A little girl hearing her mother observe to another lady that she was going in half mourning, inquired whether any of her relations were half dead?

—An honest Dutchman, on being asked how often he shaved, replied, "Dree dimes a week every tay but Soontay; den I shafe every tay.

—"Well, Patrick, are you digging a hole?" No yere honor, I'm digging out the dirt, and leaving the hole there.

—"Father, is all them old fellers alive yet?" Who do you mean my child" "Why! Moses and Abram and Deuteronomy and all them."

—There now! we've repeated in our old age, what we used to pride ourselves in doing in the early years of our practice, giving half a dozen pills at a single swallow, when the patient would persist there was but one; it was a knack we had; now, we've done better; we've given our patient reader half a hundred pills of laugh, and it did'nt occur to him that he had taken any at all, until now that his attention is called to the fact; laugh-provoking, fun-promoting pills, worth more than a shop full of druggery, especially in these lazy, weary, sleepy days of July; when long articles can't be comprehended and only act as opiates. Little does the thoughtless reader imagine what a valuable medicine he gets "thrown in," with his newspaper, the editor of which gives him weekly, half a column of fun provoking absurdities and laughable realities; more health promoting that half column of humor than any "potion" ever sold over the apothecary's counter; more life giving, than any brandy smash, gin sling or whiskey toddy ever swallowed! and in their gratitude, delinquent readers should, on the spot, go and pay up all newspaper arrearages, and advance for the next two years; for little do they know how much they are indebted to editors who conoct all these funny things, and circulate them among one another for the general good; for fun is the best physic, the world over; in the light of these truths who shall not say that Artemus Ward is not the best Doctor of our day? his mediicine is so easy to take too. Go on Arty, you're a good fellow.

